# Naoluo Xintong Decoction in the Treatment of Ischemic Stroke: A Network Analysis of the Mechanism of Action

**DOI:** 10.3389/fphar.2022.809505

**Published:** 2022-05-20

**Authors:** Ni Wang, Furui Chu, Changyi Fei, Lingyu Pan, Yongzhong Wang, Weidong Chen, Daiyin Peng, Xianchun Duan, Ling He

**Affiliations:** ^1^ Department of Pharmacy, The First Affiliated Hospital of Anhui University of Traditional Chinese Medicine, Hefei, China; ^2^ School of Pharmacy, Anhui University of Chinese Medicine, Hefei, China; ^3^ School of Traditional Chinese Medicine, Anhui University of Chinese Medicine, Hefei, China

**Keywords:** network analysis, Naoluo Xintong decoction, ischemic stroke, molecular docking, animal experiment

## Abstract

The mechanism of action of Naoluo Xintong decoction (NLXTD) for the treatment of ischemic stroke (IS) is unknown. We used network analysis and molecular docking techniques to verify the potential mechanism of action of NLXTD in treating IS. The main active components of NLXTD were obtained from the Traditional Chinese Medicine Systems Pharmacology (TCMSP) database, and IS targets were collected from the Online Mendelian Inheritance in Man (OMIM), GeneCards, and Drugbank databases; their intersection was taken. In addition, Gene Ontology and Kyoto Encyclopedia of Genes and Genomes pathway analyses were performed and used to build protein-protein interaction networks. AutoDock Vina software was used for molecular docking, and animal experiments were conducted to verify the results. Hematoxylin and eosin staining was used to observe the brain morphology of rats in each group, and real-time quantitative polymerase chain reaction (RT-qPCR) was used to detect the expression level of relative mRNA in the brain tissue of rats. Western blot was used to detect the expression level of relative protein in the brain tissue of rats. Network analysis and molecular docking results showed that CASP3, NOS3, VEGFA, TNF, PTGS2, and TP53 are important potential targets for NLXTD in the treatment of IS. RT-qPCR and western blot results showed that NLXTD inhibited the expression of CASP3, TNF, PTGS2, and TP53 and promoted the expression of VEGFA and NOS3. NLXTD treats IS by modulating pathways and targets associated with inflammation and apoptosis in a multicomponent, multitarget manner.

## Introduction

Naoluo Xintong decoction (NLXTD) is a classical herbal prescription of Xin’an medicine. Xin’an medicine, one of the most important academic schools of Traditional Chinese Medicine (TCM), is originated from Song Dynasty and flourished in Ming and Qing Dynasties. NLXTD is an impartial part of Xin’an medicine, has profound and rich clinical experiences in the treatment of cardio-cerebrovascular disease, especially ischemic stroke (He and Wang, 2017; He et al., 2019). However, we do not know why NLXTD is effective in treating ischemic stroke (IS), so we are curious about what is the mechanism of action of NLXTD for IS. Cerebral apoplexy, also called cerebral infarction, refers to cerebral artery stenosis caused by various reasons or even complete or incomplete obstruction, resulting in blood circulation disorders in the brain that lead to brain tissue ischemia and hypoxic necrosis at the site of obstruction; then, corresponding body function defects occur (Campbell et al., 2019). The disease, which often leaves its victims disabled, comprises two types: ischemic stroke and hemorrhagic stroke. Among these two, the IS incidence rate is usually higher than that of hemorrhagic stroke, accounting for approximately 80% of all infarctions (Zhao, 2019).

NLXTD is a well-known formula used clinically by Wang Le Tao for ischemic cerebrovascular diseases (He et al., 2019). It consists of *Hedysarum Multijugum Maxim* (HQ), *Panax Notoginseng F. H. Chen Ex C. Chow* (SQ), *Carthami Flos* (HH), *Gastrodia elata Bl* (TM), *Angelicae Sinensis Radix* (DG), *Chuanxiong Rhizoma* (CX) and *Scolopendra subspinipes* (WG). NLXTD promotes blood circulation and removes blood stasis (Hong et al., 2021). A previous study has shown that NLXTD reduces tumor necrosis factor alpha (TNF-α) protein expression in the frontoparietal cortex of reperfused rats with cerebral ischemia (Wang et al., 2018). NLXTD has promoted cerebral vascular regeneration in a rat model of middle cerebral artery occlusion (Tan et al., 2020). Because NLXTD is composed of a variety of traditional Chinese medicines and has the characteristics of multiple components and multiple targets, we used network informatics tools to explore and change the research mode of one drug, one target, and one pathway.

Network analysis uses the integration of a large number of network database resources and bioinformatics technology to build an overall network of drugs and disease targets, so as to explain the mechanism of multipathway, multitarget effects at micro and macro levels (Luo et al., 2020). Molecular docking technology uses computer simulations of ligand receptor protein interactions (Pinzi and Rastelli, 2019). We constructed a network of drugs and disease targets through network analysis, identified potential mechanisms and key targets, and used molecular docking technology for docking and calculations to screen out six genes that had the highest correlation with the disease. Then, we detected mRNA and protein expression levels of the six genes using real-time quantitative polymerase chain reaction (RT-qPCR) and western blot.

## Materials and Methods

### Potentially Active Drug Ingredients and Disease Targets

The chemical compositions of HQ, SQ, HH, CX, and DG were obtained using the Traditional Chinese Medicine Systems Pharmacology (TCMSP) database. The chemical composition of TM was collected from China National Knowledge Internet and PubMed. Two active ingredients: Drug-likeness (DL) ≥ 0.18 and Oral bioavailability (OB) ≥ 30% were selected (Campbell et al., 2019; Cui et al., 2020), and gene annotation was carried out with the UniProt database. Online Mendelian Inheritance in Man (OMIM) (Amberger et al., 2015), Drugbank (Wishart et al., 2018), and GeneCards (Xia et al., 2021) databases were searched for genes related to ischemic cerebral apoplexy. The intersection of drug target and disease target was selected as the therapeutic target of NLXTD for IS.

### Protein-Protein Interaction Network Construction

The STRING database is designed to integrate all known and predicted associations between proteins (Szklarczyk et al., 2017). The goal targets were imported into the STRING database to construct a protein-protein interaction (PPI) network and obtain PPI information for additional analysis.

### Gene Ontology and Kyoto Encyclopedia of Genes and Genomes Analyses

The goal targets were inputted to the DAVID database and Gene Ontology (GO) and Kyoto Encyclopedia of Genes and Genomes (KEGG) pathway enrichment analyses were conducted. Detailed information on pathways and key targets was downloaded from online platforms to explore potential mechanisms for treating IS.

### “Component-Target-Pathway” Network Construction

To display the association between drugs and diseases more intuitively, Cytoscape 3.6.1 softw- are was used to create a “component-target-pathway” network and a neuroinflammation subnetwork.

### Molecular Docking

The structural information of the ligand molecule was obtained from the National Center for Biotechnology Information Search (NCBI) database, and three-dimensional (3D) structures were drawn with ChemDraw 17.0 software and saved to a Mol_2_ format. Target protein crystal structures were obtained from the Protein Data Bank (PDB) database, and protein crystal structures with resolution less than 3 Å were selected to build molecular docking models (Li et al., 2021a). Docking pocket areas were predicted with the DeepSite online tool (Jiménez et al., 2017). Hydrogenation and dehydration were performed using AutoDockTools (v.1.5.6), and docking was performed using AutoDock Vina (v.1.1.2). The docking results were visualized with PyMOL (v.2.4.1) and Ligplot (v.2.2.5).

## Animal Experiments

### Establishment of Animal Models

Healthy male Sprague-Dawley rats weighing 230–270 g were purchased from the Experimental Animal Center of Anhui Medical University [SCXK (Su)2017-0003]. The rats underwent acclimatization feeding for 1 week.

On the basis of prior research (Longa et al., 1989), a small incision was cut 1 cm from the bifurcation of the common carotid artery, a fish line was inserted into the small incision approximat- ely 18–22 mm, and the embolus line was withdrawn after 2 h of ischemia; then, the wound was sutured. At 2 h postoperatively, the neurological function of each operated rat was scored according to the Longa scale as follows: 0, the rat moved normally; 1, the left front paw could not be success- fully extended; 2, the rat was placed on the platform and turned to the left when crawling; 3, the rat’s body was skewed to the left when crawling; and 4, the rat could not crawl by itself. Longa scores of 1–3 were considered appropriate for inclusion in the study.

### Grouping and Drug Administration

One hundred twenty rats were divided into the following groups (*n* = 20 per group): a sham group, an ischemia/reperfusion (I/R) group, a high-dose NLXTD group, a medium-dose NLXTD group, a low-dose NLXTD group, and a nimodipine (NMDP) positive control group. The concentrations of NLXTD were 1 g/ml, 2.5 g/ml, and 5 g/ml in the low, medium, and high dose groups by gavage, respectively, and the concentration of NMDP was 0.04 g/ml in the NMDP group by gavage. The volume of gavage was 100 g/ml. Sham and I/R groups were gavaged with equal amounts of saline for 7 days. After the last administration in each group, rats were anesthetized, and brain tissue was taken and stored in a −80°C refrigerator. NLXTD was prepared according to the method of reference (He et al., 2019). Specific drug information is shown in Supplementary Table S1. Firstly, NLXTD was mixed at the ratio of 15: 5: 3: 5: 5: 5 (HQ: CX: SQ: TM: HH: DG) and soaked for 30 min. The mixture was boiled two times with 8 folds and 6.4 folds volume deionized water for an hour, respectively. Next the first decoction was merged with the second decoction and the powdered medicine of Scolopendra was added to decoction. The decoctions were filtered, centrifuged and concentrated to 1 g/ml, 2.5 g/ml, and 5 g/ml by rotary evaporation.

### Hematoxylin and Eosin Staining

The rat brain tissue was fixed with tissue fixative; then, brain tissue was taken, dehydrated, wax-dipped, embedded, and sectioned (3–4 μm/section). Sections were dewaxed to water for hematoXylin and eosin (HE) staining before being dehydrated and sealed for microscopic examination. The examination observed morphological changes in hippocampal neurons (at ×200 high magnification field of view).

### Enzyme-Linked Immunosorbent Assay

A lysate was added at a rate of 150–250 µl of lysate/20 mg of tissue. After sufficient lysis, the samples were centrifuged at 10,000–14,000 g for 3–5 min, and the supernatant was removed. The protein concentration of the sample was measured, and TNF-α was detected according to the procedure described in the packaging for the enzyme-linked immunosorbent assay kit (Shanghai Jianglai Bio, China).

### RT-qPCR

Rat brain tissue was collected, total RNA was extracted, and the RNA was reverse transcribed to cDNA for the PCR reaction. A relative quantitative analysis was performed using the 2^−∆∆Ct^ method; the primer sequences are shown in Supplementary Table S2.

### Western Blotting

Rat brain tissue was extracted, and the protein concentration was calculated by Bicinchoninic Acid Assay (BCA). Then, SDS gel electrophoresis was started, followed by membrane transfer, antigen blocking, and primary antibody incubation [with anti-GAPDH (Proteintech, 60004-1-Ig, United States), anti-Caspase3 (Proteintech, 66470-2-Ig, United States), anti-NOS3 (Proteintech, 27120-1-AP, United States), anti-VEGFA (Proteintech, 66828-1-Ig, United States), anti-TNFa (Proteintech, 60291-1Ig,United States), anti PTGS2 (Proteintech, 12375-1-AP, United States), anti-TP53 (Proteintech, 60283-2-Ig, United States)]. Then, secondary antibody incubation [with peroxidase-conjugated goat anti-mouse IgG (H + L) 1:10,000 and horseradish-peroxidase-conjugated rabbit anti-human IgG (H + L) 1:10,000] was performed.

### Statistical Analysis

SPSS 20.0 software was used to analyze the data. Means ± standard deviations were used for normally distributed measures, and one-way analysis of variance was used for multiple data groups. *p* < 0.05 was statistically significant.

## Results

### Screening of Potentially Therapeutic Targets of NLXTD for IS

**FIGURE 1:**
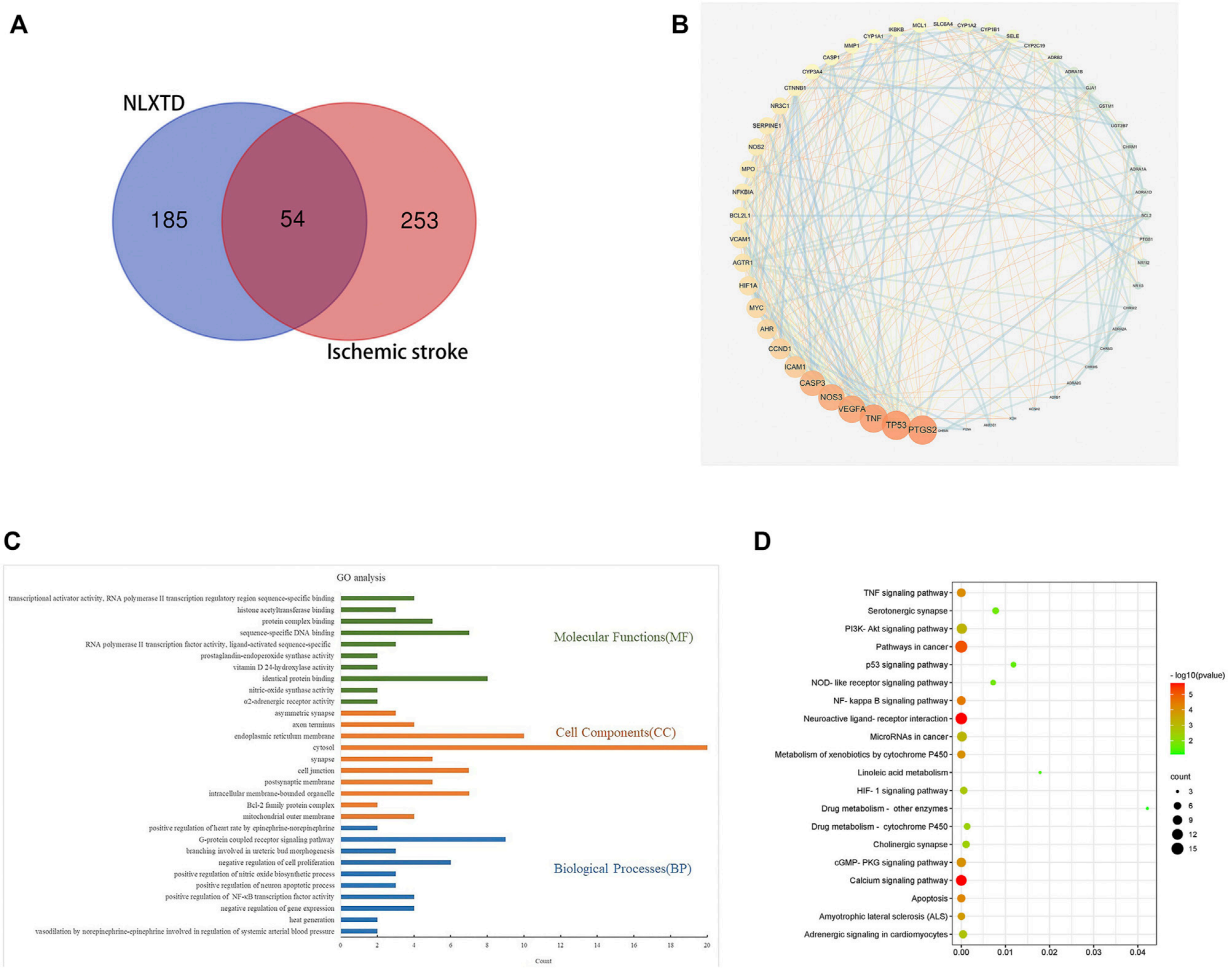
VENN **(A)**, PPI network **(B)**, GO **(C)**, and KEGG **(D)** analysis of NLXTD.

A total of 64 NLXTD components with DL ≥ 0.18 and OB ≥ 30% were identified, as shown in Supplementary Table S3 **.** Results included 20 components of HQ, 8 components of SQ, 22 components of HH, 5 components of TM, 2 components of DG, and 7 components of CX. Overall, 239 potential targets were obtained by removing duplicate values of these identified components. A total of 307 genes related to ischemic cerebral apoplexy were obtained from OMIM, GeneCards, and Drugbank databases after aggregation and deduplication. The disease targets were intersected with the potential targets of NLXTD, and 54 of them (Figure 1A), including NOS2, PTGS1, PTGS2, and CHRM2, were obtained as common targets. These common targets were considered the potential targets of NLXTD for the treatment of IS.

### Protein-Protein Interaction Network Analysis

The 54 targets were inputted into the STRING database to build a PPI network. In the network, a darker node represented a larger value of the degree. The median value greater than twice the Degree value was selected as the screening basis to obtain six core objectives: CASP3, NOS3, VEGFA, TNF, PTGS2, and TP53 (Figure 1B).

### Functional Enrichment Analysis and Pathway Enrichment Analysis

GO (*p* < 0.01) functional enrichment results showed that NLXTD treatment IS was associated with 97 biological processes mainly involving vasodilation and systemic arterial blood pressure regulation by norepinephrine, the nitric oxide biosynthesis process, and negative regulation of gene expression; in addition, 14 cellular compositions involving cell junctions, synapses, and endoplasm- ic reticulum membranes and 33 molecular functions related to enzyme activity, identical protein binding, and protein complex binding were noted. The top 10 results by *p* value were used to build a GO enrichment analysis histogram (Figure 1C).

Analysis of the enrichment results of KEGG (*p* < 0.05) pathway yielded 52 pathways. Twenty signaling pathways that may be neurologically related to cerebral ischemic cells were selected for mapping (Figure 1D). Analysis of the enrichment results showed that drug metabolism-related pathways involved linoleic acid metabolism, drug metabolism and other enzymes, drug metabolism and cytochrome P450, and more; regulation of cell-related pathways involved the PI3K-Akt signaling pathway, apoptosis, and the calcium signaling pathway. Neural-related pathways involved the TNF-α signaling pathway and neuroactive ligand receptor interactions. The results suggest that NLXTD exerts its therapeutic effects through drug metabolism, cellular modulation, and nerve repair.

### “Component-Target-Pathway” Network Analysis

**FIGURE 2:**
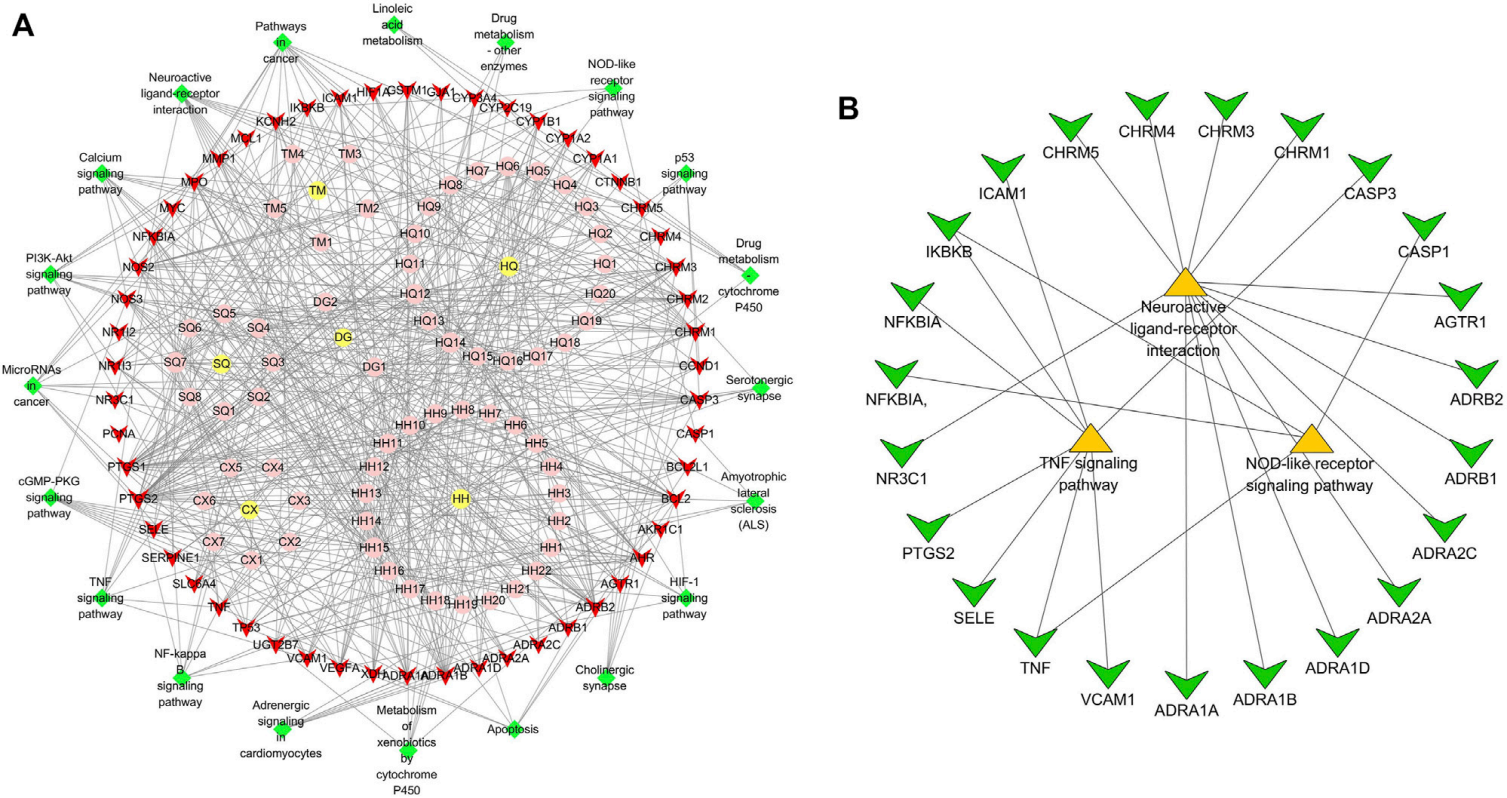
“Component-target-pathway” network **(A)** and “neuroinflammatory-pathway-target” sub-network **(B)** analysis of NLXTD.

The 64 components and 54 targets of NLXTD therapies for IS and the 20 possible pathways related to ischemic stroke analyzed by KEGG enrichment were imported into Cytoscape 3.6.1 software to construct a component target pathway network map (Figure 2A). In the map, red represents the common target, green represents the pathway, yellow represents the drug, and pink represents the active ingredient of the drug; the lines are the interactions between these variables. KEGG enrichment results showed three inflammation-related pathways. A neuroinflammatory pathway target subnetwork (Figure 2B) was constructed using Cytoscape 3.6.1 software, which identified 23 targets, including three core targets: CASP3, TNF, and PTGS2.

### Molecular Docking Validation Analysis

**FIGURE 3:**
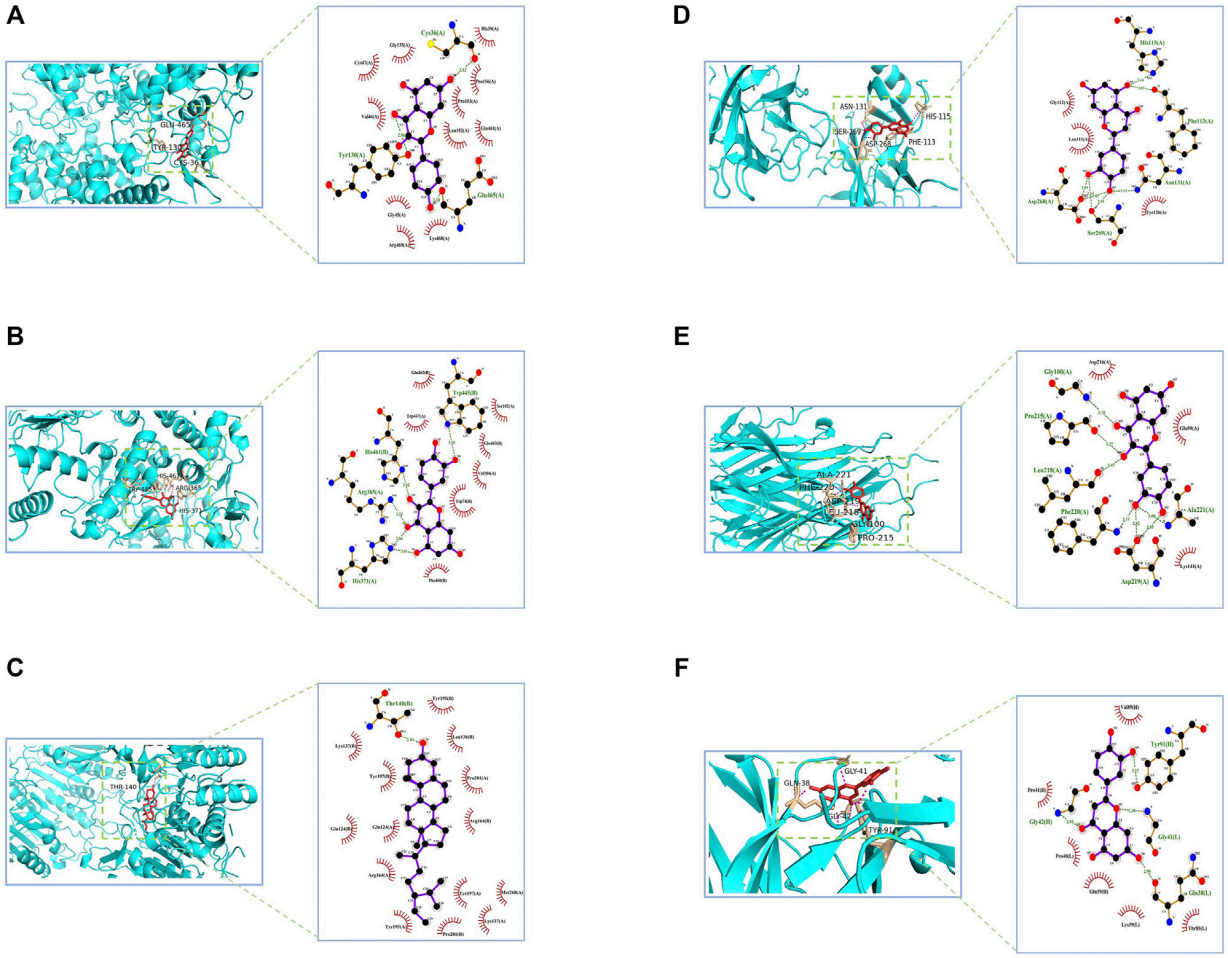
CASP3, NOS3, VEGFA, TNF, PTGS2, and TP53 molecular docking diagram. Notes: PTGS2- kaempferol **(A)** NOS3- quercetin **(B)** CASP3- beta-sitosterol **(C)** TP53- luteolin **(D)** TNF- quercetin **(E)** VEGFA- luteolin **(F)**.

The potentially active ingredients in NLXTD were obtained by reverse screening of CASP3, NOS3, VEGFA, TNF, PTGS2, and TP53; 27 components, including kaempferol, calycosin, and baicalein, were obtained (Supplementary Table S4). Crystal structures of key target proteins were obtained from the PDB database: CASP3 (3DEK), NOS3 (1M9J), VEGFA (7KEZ), TNF (7JRA), PTGS2 (5F1A), and TP53 (2VUK). Using DeepSite to predict possible docking pockets for target proteins, AutoDock Vina performed docking and calculated binding affinity, with the binding region restricted to the DeepSite-predicted docking pockets. The docking results are shown in Figure 3, in which the hydrogen bonds are represented as dashed lines. The accepted binding affinity (kcal/mol) value of less than −5.0 kcal/mol indicates good binding activity for the docking mode, and the accepted value of less than −7.0 kcal/mol indicates strong binding activity (Supplementary Table S4) (Hsin et al., 2013; Campbell et al., 2019).

### Neurological Functional Scores

**FIGURE 4:**
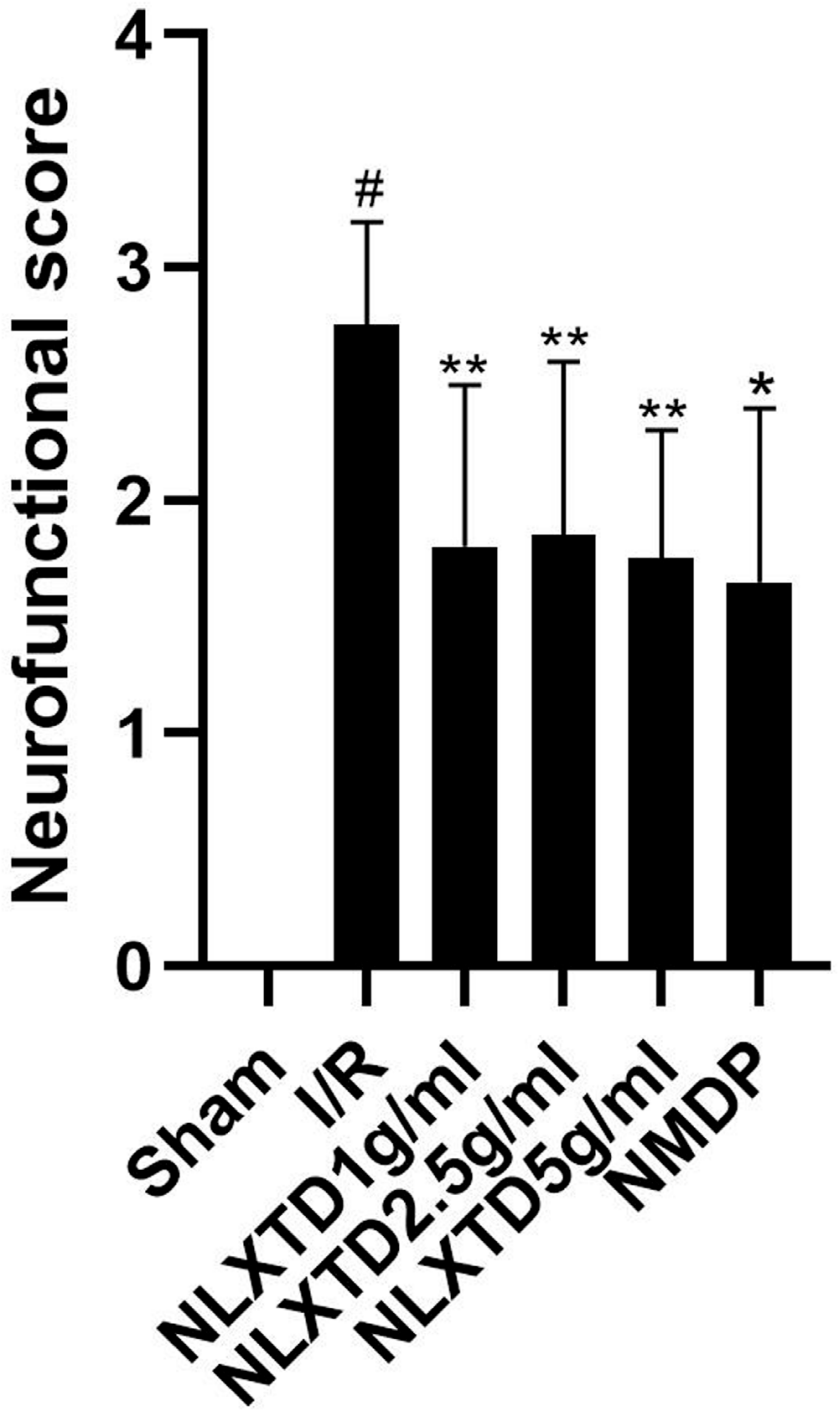
Neurofunctional score analysis of NLXTD. Notes: ^#^ *p* < 0.01 vs. Sham group; *p < 0.01, **p < 0.05 vs. I/R group.

The results of neurological functional scores are shown in Figure 4. The Longa score of rats in the I/R group increased compared with the scores of rats in the sham group (*p* < 0.01), which shows that the model was successful. The Longa score of rats in the high-, medium-, and low-dose NLXTD groups and in the NMDP group decreased compared with the I/R group (*p* < 0.05 and *p* < 0.01 for each NLXTD and for NMDP, respectively).

### Hematoxylin and Eosin Staining Assessment

**FIGURE 5:**
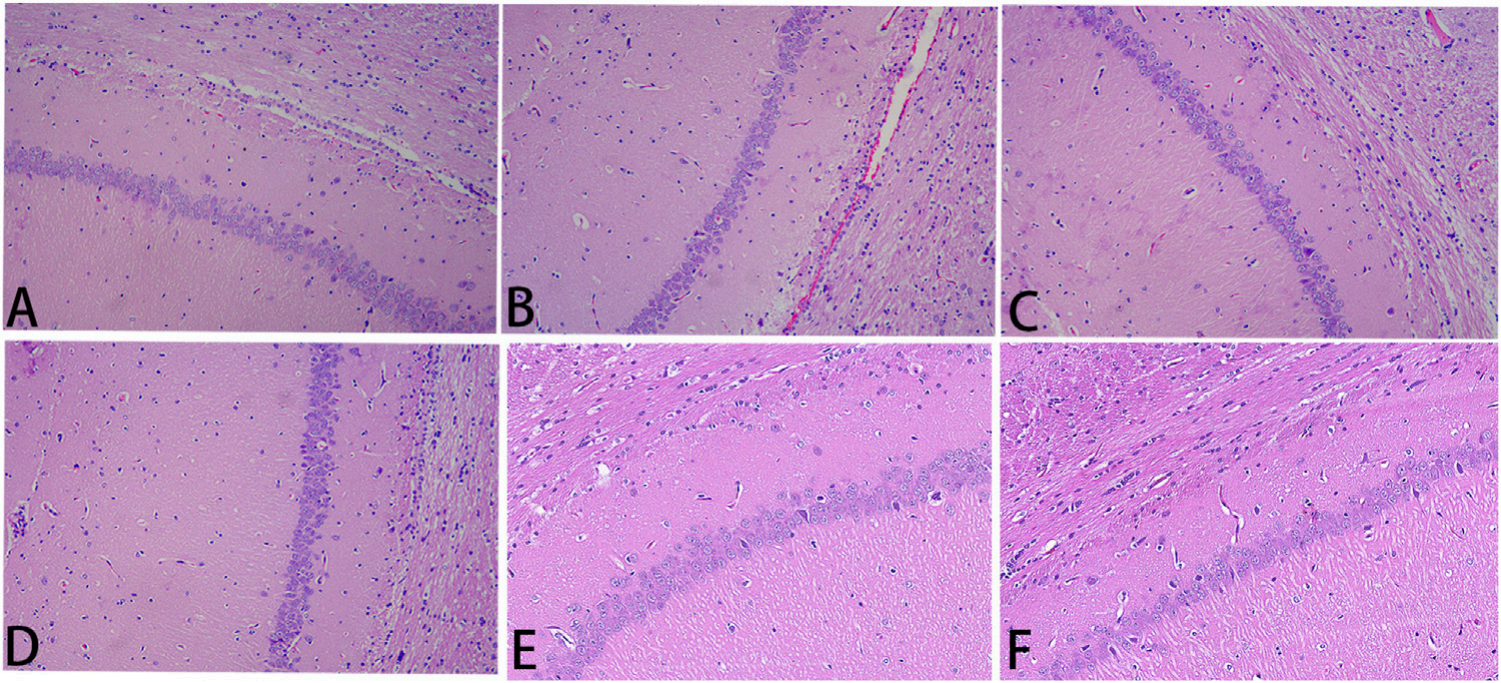
He staining. Notes: **(A)** sham; **(B)** I/R; **(C)** NMDP; **(D)** NLXTD1 g/ml; **(E)** NLXTD2.5 g/ml; **(F)** NLXTD5 g/ml.

Results of HE staining, which detected the degree of histopathological damage in the brain of each group of rats, are shown in Figure 5. In the sham group, the brain tissue was clearly structured, with neat and dense cell arrangement and normal morphology; in the I/R group, the neuronal cells were deformed and atrophied, with deeply stained cytosol and disorganized brain tissue texture, showing obvious pathological damage. Compared with the damage in the I/R group, the degree of pathological damage was significantly improved in the high-dose NLXTD group as well as in the medium- and low-dose groups and the NMDP group.

### Naoluo Xintong Decoction Inhibits Tumor Necrosis Factor-α Inflammatory Factor

**FIGURE 6:**
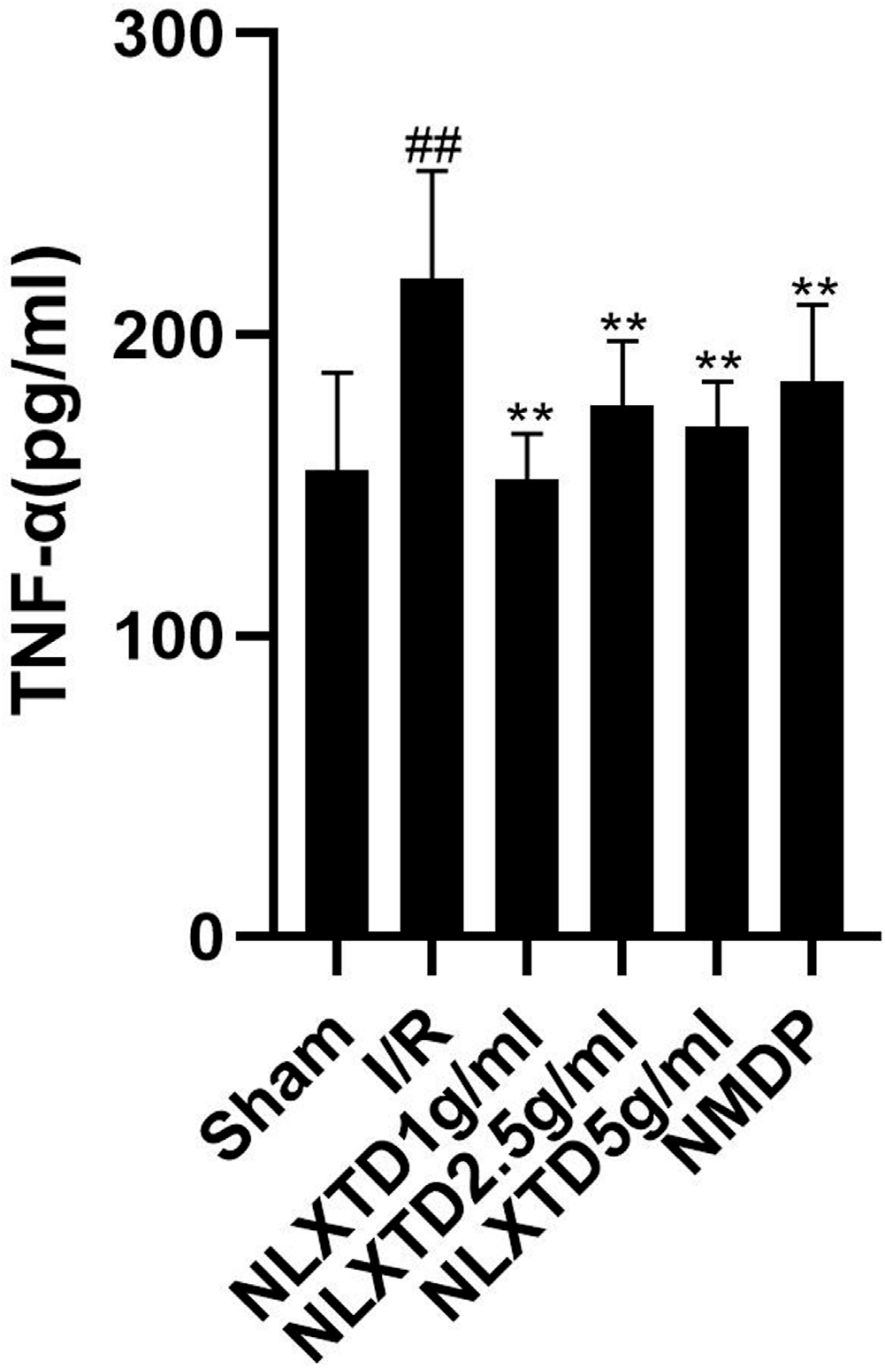
Elisa analysis of NLXTD. Notes: ^##^ *p* < 0.05 vs. Sham group; **p < 0.05 vs. I/R group.

The results of TNF-α indicators are shown in Figure 6. The TNF-α content was higher in the I/R group than in the sham group (*p* < 0.05). Compared with the content level in the I/R group, TNF-α content was lower in the high-dose NLXTD group as well as in the medium- and low-dose groups and the NMDP group (*p* < 0.05 for all comparisons).

### Expression of CASP3, NOS3, VEGFA, TNF, PTGS2, and TP53 mRNA

**FIGURE 7:**
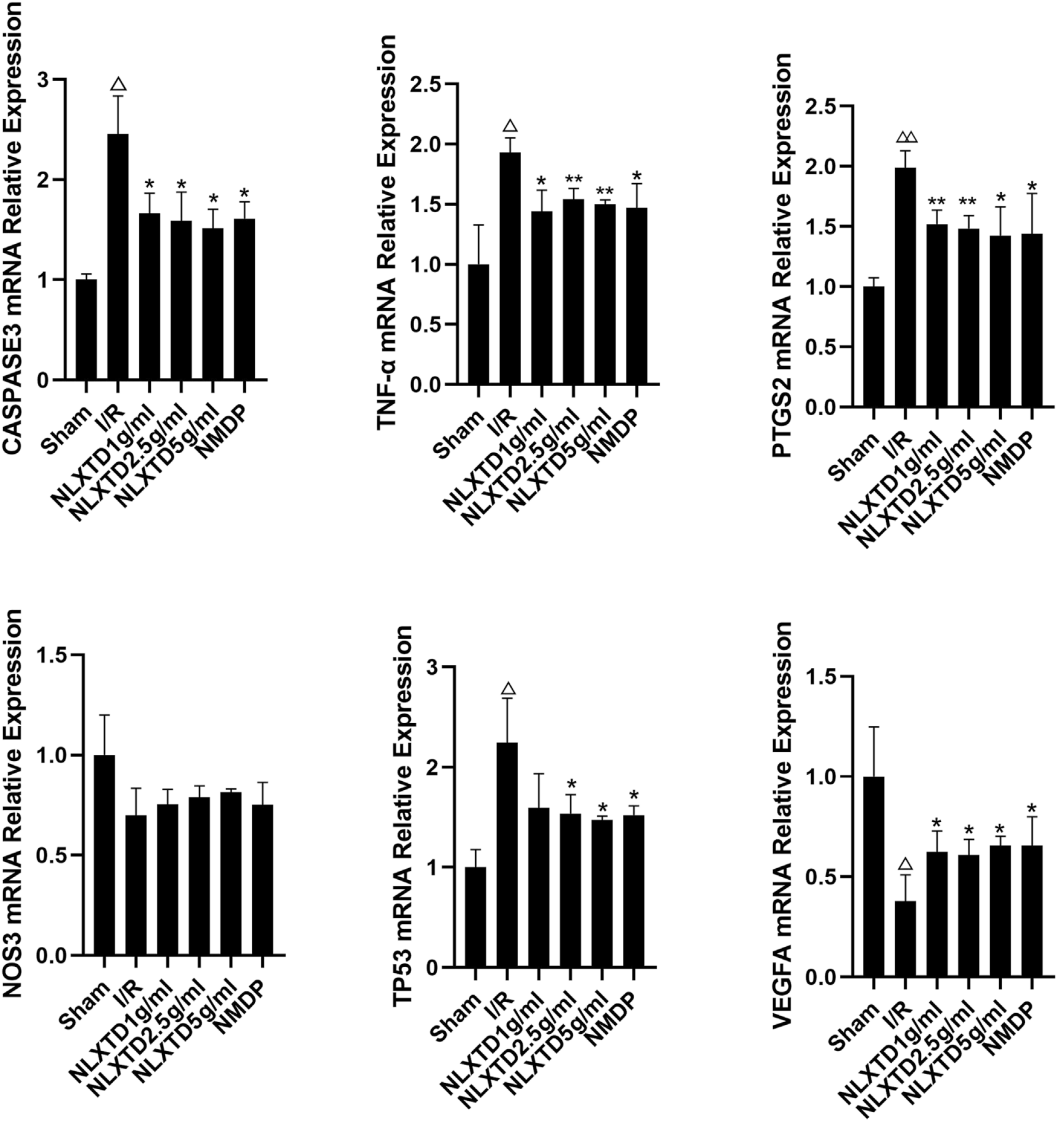
mRNA expression of CASP3, TNF, PTGS2, TP3, NOS3 and VEGFA. Notes: ^△△^ *p* < 0.01, ^△^ *p* < 0.05 vs. Sham group; **p < 0.01, *p < 0.05 vs. I/R group (*n* = 3).

CASP3, TNF, PTGS2, and TP53 mRNA in the I/R group showed a trend of increasing quantities compared with the sham group (*p* < 0.01, *p* < 0.05, See Figure 7 for details). Compared with the sham group levels, NOS3 mRNA decreased in the I/R group, but not significantly (*p* > 0.05), and VEGFA mRNA showed a trend toward decreasing levels (*p* < 0.05). Compared with levels in the I/R group, TP53, CASP3, TNF, and PTGS2 mRNA showed a trend of decreasing quantities in the high-, medium-, and low-dose NLXTD groups and in the NMDP group (*p* < 0.01,*p* < 0.05, Figure 7 for details), but TP3 mRNA was not significantly different in the NLXTD low-dose group (*p* > 0.05). Compared with levels in the I/R group, VEGFA mRNA showed a trend of increasing quantities in the high-, medium-, and low-dose NLXTD groups and in the NMDP group (*p* < 0.05) and NOS3 mRNA increased but not significantly (*p* > 0.05) (Figure 7). Thus, NLXTD decreased the expression of CASP3, TNF, and PTGS2 mRNA, increased the expression of VEGFA mRNA, and promoted the expression of NOS3 and TP3 mRNA.

### Expression of CASP3, NOS3, VEGFA, TNF, PTGS2, and TP53 Proteins

**FIGURE 8:**
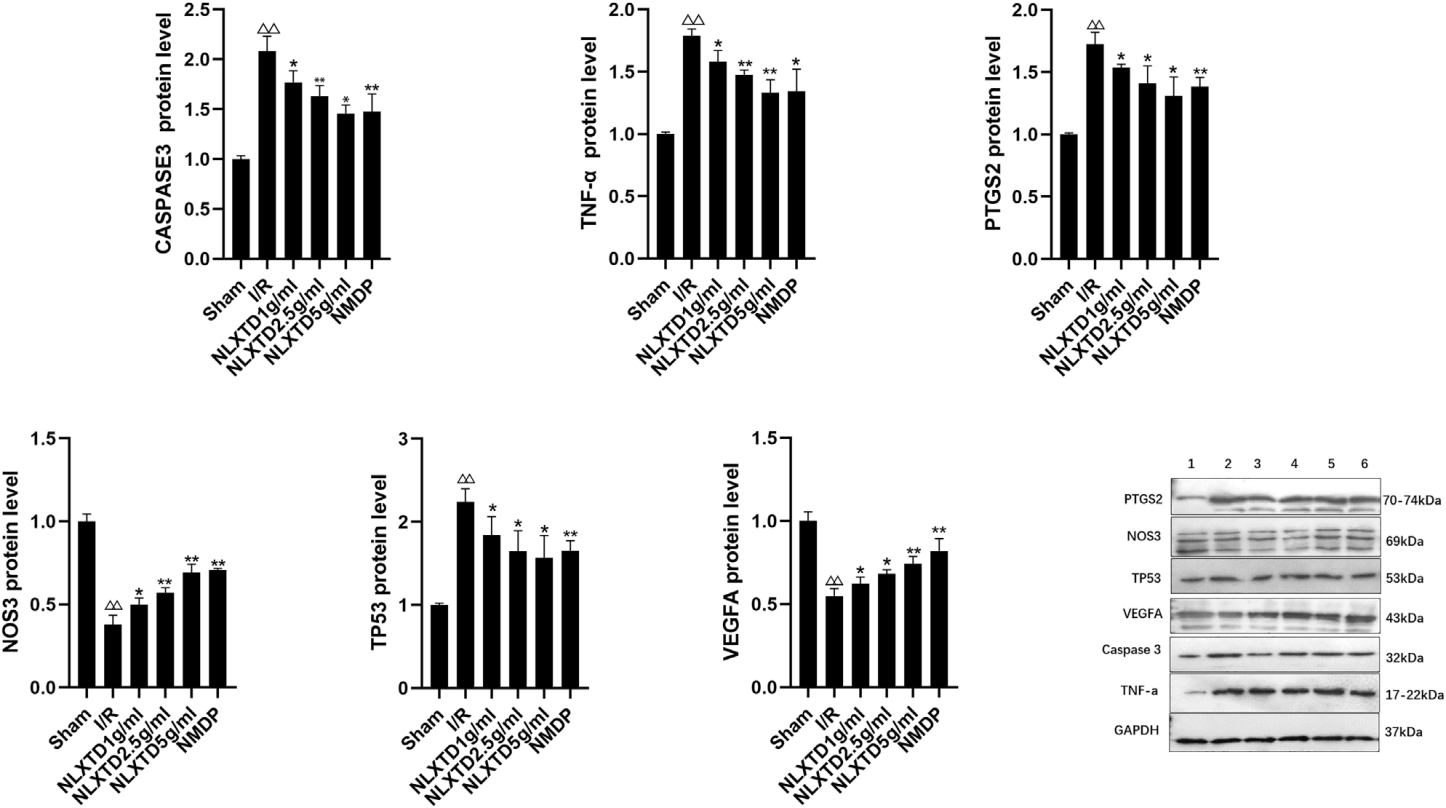
Protein expression of CASP3, TNF, PTGS2, TP3, NOS3 and VEGFA. Notes: ^△△^ *p* < 0.01 vs. Sham group; **p < 0.01, *p < 0.05 vs. I/R group. 1: Sham, 2: I/R, 3: NLXTD5 g/ml, 4:NLXTD2.5 g/ml, 5: NLXTD1 g/ml, 6: NMDP (*n* = 3).

CASP3, TNF, PTGS2, and TP3 proteins were significantly increased (*p* < 0.01) and NOS3 and VEGFA proteins were significantly decreased (*p* < 0.01) in the I/R group compared with the sham group. Compared with levels in the I/R group, CASP3, TNF, PTGS2, and TP3 proteins were significantly decreased (*p* < 0.01,*p* < 0.05, Figure 8 for details) and NOS3 and VEGFA proteins were significantly increased (*p* < 0.01,*p* < 0.05, See Figure 8 for details) in the high-, medium-, and low-dose NLXTD groups and in the NMDP group. NLXTD decreased CASP3, TNF, PTGS2, and TP3 protein levels and increased NOS3 and VEGFA protein levels.

## Discussion

IS can block blood flow to the brain, so the brain tissue undergoes ischemia and hypoxia as well as softening or even necrosis, which seriously affects the patient’s standard of living. NLXTD adheres to the theory of traditional Chinese medicine and integrates its medicinal effects from various aspects. In this study, 64 drug components, 239 drug targets, and 307 disease targets of NLXTD were screened by network analysis. The PPI network, the component target pathway network, and the neuroinflammatory subnetwork indicated drug-target interactions. KEGG results showed a total of 52 pathways, among which the largest number of enriched genes were in the calcium signaling pathway and the PI3K-Akt pathway. The calcium signaling pathway is inseparably related to the anti-ischemic injury effect (Liu et al., 2017). It has been suggested that activated Akt can prevent apoptosis and promote cell survival; thus, activating the PI3K-Akt signaling channel can alleviate IS (Zheng et al., 2019).

IS may trigger complex cellular events that lead to neuronal apoptosis in a progressive manner (Zhang et al., 2019). TP53 is an important apoptosis-related gene involved in the apoptosis initiation process and has an important role in regulating cell growth, maintenance, apoptosis, and DNA repair progression (Wei et al., 2019). When IS occurs, p53 expression levels are rapidly upregulated, which directly disrupts the permeability of the mitochondrial membrane by enhancing the Bcl-2 family of proapoptotic proteins (PUMA and BAX), thereby damaging cells in the ischemic semidark zone (Lu et al., 2020). Apoptosis in ischemic neurons is mainly driven by Bcl-2 family genes and caspase family genes. Caspases are a group of cysteine aspartate proteases, and CASP3 is an important component of apoptosis that plays a major role in the onset and development of apoptosis. When IS occurs, activated CASP3 causes chromatin condensation as well as DNA breakage, ultimately leading to cell death (Li et al., 2000). The neuroinflammatory response after stroke plays an important role in regulating the survival of neurons, and inflammation regulation is the key to treating stroke (Sotomayor-Sobrino et al., 2019). The neuroinflammatory response is closely related to the activation and polarization of microglia in the brain, which are rapidly activated after cerebral ischemia and hypoxia (Qin et al., 2019). The activated microglia differentiate into different phenotypes, and each regulates central nervous system homeostasis in different ways. Inflammatory cytokines include TNF-α, interleukin (IL)-1β, and cyclo-oxygenase 2 (Hanna and Frangogiannis, 2020). Studies have shown that Prostaglandin-endoperoxide synthase 2 (PTGS2) is present as an important inflammatory mediator throughout the early stages of inflammation and into inflammati- on formation; PTGS2 is an important mediator for the induction of the inflammatory cascade response in IS (Dong et al., 2019). Its overexpression can disrupt the balance of the internal environment, participate in the inflammatory response after brain injury, and promote the expansion of cerebral infarct size (Zhou et al., 2019). TNF is a tumor necrosis factor and TNF-α has the ability to damage the blood brain barrier, promote coagulation, and induce hypoxia tolerance and nerve growth factor production, which can promote spontaneous recovery of IS neuronal function (Chen et al., 2019a). NOS3, also known as endothelial nitric oxide synthase (eNOS), is mainly involved in regulating angiogenesis and inhibiting platelet coagulation as well as inhibiting leukocyte adhesion and other important physiological processes through the production of nitric oxide (Sun et al., 2016), which can diastole blood vessels and prevent thrombosis. VEGF is an endothelial growth factor that can promote endothelial cell proliferation, increase vascular permeability, and promote the formati- on of capillaries. The hypoxia-induced VEGFA signaling pathway is closely associated with ischemic cerebrovascular disease, affecting atherosclerosis and intrinsic collateral circulation, and is involved in cerebral infarction pathogenesis (Li et al., 2021b). When cerebral ischemia occurs, VEGF is upregulated and promotes angiogenesis, resulting in a positive effect on brain plasticity and functional recovery (Chen et al., 2019b).

The molecular docking results showed that the potentially active components of NLXTD stably bound to the core target proteins CASP3, NOS3, VEGFA, TNF, PTGS2, and TP53 indicating that NLXTD may act on the targets of CASP3, NOS3, VEGFA, TNF, PTGS2, and TP53. The results of animal experiments showed that NLXTD treats IS by inhibiting apoptosis, controlling inflammation, and regulating angiogenesis.

## Conclusion

In summary, this experiment used network analysis and molecular docking techniques to predict the potential targets of NLXTD for the treatment of IS and validated them with animal experiments. The results suggest that NLXTD can treat IS through multitarget, multicomponent, and multipathway mechanisms.

## Data Availability

The original contributions presented in the study are included in the article/Supplementary Material, further inquiries can be directed to the corresponding authors.
