# Supplementary material for: Naoluo Xintong Decoction in the Treatment of Ischemic Stroke: A Network Analysis of the Mechanism of Action
Source: Front Pharmacol. 2022 May 20;13:809505. doi: 10.3389/fphar.2022.809505 (PMC9163544; doi:10.3389/fphar.2022.809505)
Supplement: Supplementary file 1 [file DataSheet1.docx]

**Table S1** **Constituents of NLXTD**

| **Components** | **Part used** | **Proportion** |
| --- | --- | --- |
| **Huang Qi (HQ) *(Hedysarum Multijugum Maxim)*** | Radix | 15 |
| **Chuan Xiong (CX) (*Chuanxiong Rhizoma*)** | Rhizoma | 5 |
| **San Qi (SQ) (*Panax Notoginseng (Burk.) F. H. Chen Ex C. Chow)*** | Radix et Rhizome | 3 |
| **Tian Ma (TM) (*Gastrodia elata Bl*)** | Rhizome | 5 |
| **Wu Gong (WG)**  ***(******Scolopendra subspinipes)*** | All | 2 |
| **Hong Hua (HH) (*Carthami Flos)*** | Flos | 5 |
| **Dang Gui (DG) (*Angelicae Sinensis Radix)*** | Radix | 5 |

**Table S2 Primer sequence**

| **Primer Name** | | **sequence (5'-3')** | **Product Length** |
| --- | --- | --- | --- |
| CASP3 | F | TGGACAACAACGAAACCTC | 140 |
|  | R | ACACAAGCCCATTTCAGG |  |
| NOS3 | F | GTGGTTTGCTGCCCTTG | 137 |
|  | R | GGTCCCTCATGCCAATCT |  |
| VEGFA | F | ACAGGGAAGACAATGGGA | 126 |
|  | R | CTGGAAGTGAGCCAACG |  |
| TNF-α | F | CCACCACGCTCTTCTGTC | 148 |
|  | R | GCTACGGGCTTGTCACTC |  |
| PTGS2 | F | ACTCTATCACTGGCATCCG | 113 |
|  | R | GAGCAAGTCCGTGTTCAAG |  |
| TP53 | F | TCCGGTCAGTTGTTGGA | 121 |
|  | R | GCAGAGTGGAGGAAATGG |  |
| beta-Actin | F | CCTCACTGTCCACCTTCCA | 120 |
|  | R | GGGTGTAAAACGCAGCTCA |  |

**Table S3 Potential active ingredients of NLXTD**

| **MOL ID** | **Molecule Name** | **OB/%** | **DL** | **Herb name** |
| --- | --- | --- | --- | --- |
| MOL000211 | Mairin | 55.38 | 0.78 | HQ |
| MOL000239 | Jaranol | 50.83 | 0.29 | HQ |
| MOL000296 | hederagenin | 36.91 | 0.75 | HQ |
| MOL000033 | (3S，8S，9S，10R，13R，14S，17R)-10，13-dimethyl-17-[(2R，5S)-5-propan-2-yloctan-2-yl]-2，3，4，7，8，9，11，12，14，15，16，17-dodecahydro-1H-cyclopenta[a]phenanthren-3-ol | 36.23 | 0.78 | HQ |
| MOL000354 | isorhamnetin | 49.60 | 0.31 | HQ |
| MOL000371 | 3，9-di-O-methylnissolin | 53.74 | 0.48 | HQ |
| MOL000374 | 5'-hydroxyiso-muronulatol-2'，5'-di-O-glucoside | 41.72 | 0.69 | HQ |
| MOL000378 | 7-O-methylisomucronulatol | 74.69 | 0.30 | HQ |
| MOL000379 | 9，10-dimethoxypterocarpan-3-O-β-D-glucoside | 36.74 | 0.92 | HQ |
| MOL000380 | (6aR，11aR)-9，10-dimethoxy-6a，11a-dihydro-6H-benzofurano[3，2-c]chromen-3-ol | 64.26 | 0.42 | HQ |
| MOL000387 | Bifendate | 31.10 | 0.67 | HQ |
| MOL000392 | formononetin | 69.67 | 0.21 | HQ |
| MOL000398 | isoflavanone | 109.99 | 0.30 | HQ |
| MOL000417 | Calycosin | 47.75 | 0.24 | HQ |
| MOL000422 | kaempferol | 41.88 | 0.24 | HQ,HH |
| MOL000433 | FA | 68.96 | 0.71 | HQ,CX |
| MOL000438 | (3R)-3-(2-hydroxy-3，4-dimethoxyphenyl)chroman-7-ol | 67.67 | 0.26 | HQ |
| MOL000439 | isomucronulatol-7，2'-di-O-glucosiole | 49.28 | 0.62 | HQ |
| MOL000442 | 1，7-Dihydroxy-3，9-dimethoxy pterocarpene | 39.05 | 0.48 | HQ |
| MOL000098  MOL001494  MOL001792  MOL002879  MOL000358 | Quercetin  Mandenol  DFV  Diop  beta-sitosterol | 46.43  42.00  32.76  43.59  36.91 | 0.28  0.19  0.18  0.39  0.75 | HQ,SQ,HH  SQ,CX,TM  SQ  SQ,TM  SQ,HH,DG |
| MOL000449 | Stigmasterol | 43.83 | 0.76 | SQ,HH,DG |
| MOL005344 | ginsenoside rh2 | 36.32 | 0.56 | SQ |
| MOL007475 | ginsenoside f2 | 36.43 | 0.25 | SQ |
| MOL001771 | poriferast-5-en-3beta-ol | 36.91 | 0.75 | HH |
| MOL002680 | Flavoxanthin | 60.41 | 0.56 | HH |
| MOL002694 | 4-[(E)-4-(3，5-dimethoxy-4-oxo-1-cyclohexa-2，5-dienylidene)but-2-enylidene]-2，6-dimethoxycyclohexa-2，5-dien-1-one | 48.47 | 0.36 | HH |
| MOL002698 | lupeol-palmitate | 33.98 | 0.32 | HH |
| MOL002706 | Phytoene | 39.56 | 0.50 | HH |
| MOL002707 | phytofluene | 43.18 | 0.50 | HH |
| MOL002710 | Pyrethrin II | 48.36 | 0.35 | HH |
| MOL002712 | 6-Hydroxykaempferol | 62.13 | 0.27 | HH |
| MOL002714 | baicalein | 33.52 | 0.21 | HH |
| MOL002717 | qt_carthamone | 51.03 | 0.20 | HH |
| MOL002719 | 6-Hydroxynaringenin | 33.23 | 0.24 | HH |
| MOL002721 | quercetagetin | 45.01 | 0.31 | HH |
| MOL002757 | 7，8-dimethyl-1H-pyrimido[5，6-g]quinoxaline-2，4-dione | 45.75 | 0.19 | HH |
| MOL002773 | beta-carotene | 37.18 | 0.58 | HH |
| MOL002776 | Baicalin | 40.12 | 0.75 | HH |
| MOL002695 | lignan | 43.32 | 0.65 | HH |
| MOL000006 | luteolin | 36.16 | 0.25 | HH |
| MOL000953 | CLR | 37.87 | 0.68 | HH,TM |
| MOL002135 | Myricanone | 40.60 | 0.51 | CX |
| MOL002140 | Perlolyrine | 65.95 | 0.27 | CX |
| MOL002151 | senkyunone | 47.66 | 0.24 | CX |
| MOL002157 | wallichilide | 42.31 | 0.71 | CX |
| MOL000359 | sitosterol | 36.91 | 0.75 | CX |
| MOL001506 | Supraene | 33.55 | 0.42 | TM |
| MOL002045 | Stigmasterol | 43.41 | 0.76 | TM |

**Table S4 Molecular docking results of CASP3, NOS3, VEGFA, TNF, PTGS2, TP53 and their reverse screened chemical components**

| MOL ID | Ingredient name | Target | Binding energy /kcal·mol^-1^ |
| --- | --- | --- | --- |
| MOL000422 | kaempferol | PTGS2 | -9.4 |
| MOL002714 | baicalein | PTGS2 | -9.4 |
| MOL000417 | Calycosin | PTGS2 | -9.4 |
| MOL001792 | DFV | PTGS2 | -9.2 |
| MOL000392 | formononetin | PTGS2 | -9.1 |
| MOL002712 | 6-Hydroxykaempferol | PTGS2 | -9.1 |
| MOL000239 | Jaranol | PTGS2 | -9.1 |
| MOL002721 | quercetagetin | PTGS2 | -9.1 |
| MOL000006 | luteolin | PTGS2 | -8.8 |
| MOL000358 | beta-sitosterol | PTGS2 | -8.6 |
| MOL002757 | 7，8-dimethyl-1H-pyrimido[5，6-g]quinoxaline-2，4-dione | PTGS2 | -8.6 |
| MOL000379 | 9，10-dimethoxypterocarpan-3-O-β-D-glucoside | PTGS2 | -8.5 |
| MOL005344 | ginsenoside rh2 | PTGS2 | -8.3 |
| MOL000442 | 1，7-Dihydroxy-3，9-dimethoxy pterocarpene | PTGS2 | -8.1 |
| MOL000371 | 3，9-di-O-methylnissolin | PTGS2 | -8.1 |
| MOL002140 | Perlolyrine | PTGS2 | -8.1 |
| MOL000380 | (6aR，11aR)-9，10-dimethoxy-6a，11a-dihydro-6H-benzofurano[3，2-c]chromen-3-ol | PTGS2 | -8.0 |
| MOL002135 | Myricanone | PTGS2 | -8.0 |
| MOL002694 | 4-[(E)-4-(3，5-dimethoxy-4-oxo-1-cyclohexa-2，5-dienylidene)but-2-enylidene]-2，6-dimethoxycyclohexa-2，5-dien-1-one | PTGS2 | -7.4 |
| MOL000098 | quercetin | PTGS2 | -7.3 |
| MOL000296 | hederagenin | PTGS2 | -7.0 |
| MOL002157 | wallichilide | PTGS2 | -6.9 |
| MOL000449 | Stigmasterol | PTGS2 | -6.6 |
| MOL000378 | 7-O-methylisomucronulatol | PTGS2 | -6.6 |
| MOL002717 | qt_carthamone | PTGS2 | -6.4 |
| MOL001494 | Mandenol | PTGS2 | -5.6 |
| MOL000098 | quercetin | NOS3 | -9.7 |
| MOL000358 | beta-sitosterol | CASP3 | -9.6 |
| MOL000006 | luteolin | CASP3 | -9.0 |
| MOL005344 | ginsenoside rh2 | CASP3 | -7.9 |
| MOL002773 | beta-carotene | CASP3 | -7.6 |
| MOL000422 | kaempferol | CASP3 | -7.4 |
| MOL000098 | quercetin | CASP3 | -7.2 |
| MOL000006 | luteolin | TP53 | -7.1 |
| MOL002714 | baicalein | TP53 | -7.1 |
| MOL000098 | quercetin | TP53 | -6.3 |
| MOL000098 | quercetin | TNF | -6.7 |
| MOL000006 | luteolin | TNF | -6.6 |
| MOL000422 | kaempferol | TNF | -6.4 |
| MOL005344 | ginsenoside rh2 | TNF | -6.3 |
| MOL000006 | luteolin | VEGFA | -7.9 |
| MOL002773 | beta-carotene | VEGFA | -7.0 |
| MOL000098 | quercetin | VEGFA | -6.3 |
